# Loneliness around the world: patterns, predictors, and well-being implications

**DOI:** 10.1007/s10654-026-01424-z

**Published:** 2026-06-26

**Authors:** David Leblang, Dennis Wesselbaum

**Affiliations:** 1 Department of Politics and Batten School of Public Policy, University of Virginia, Charlottesville, VA USA; 2 Department of Economics, University of Otago, Dunedin, New Zealand

**Keywords:** Global health, Loneliness, Subjective well-being, Survey data

## Abstract

Loneliness is increasingly recognized as a global public health concern, yet cross-national evidence remains limited. Using data from the 2023–2024 Gallup World Poll, this study provides a globally representative description of loneliness across 148 countries (*N* = 218,048). Over one in five adults reported feeling lonely, with prevalence highest in low-income countries and Sub-Saharan Africa. Multivariate analyses identified several predictors of lower loneliness, such as better health, higher education, larger household size, and employment, though their associations varied by context. Loneliness was also associated with lower subjective well-being, more frequent negative emotions, and stronger migration intentions. These patterns were most pronounced in high-income and more developed regions, where loneliness appeared more psychologically burdensome despite lower prevalence. Overall, the findings highlight loneliness as a universal but context-sensitive experience, suggesting the need for policies that consider both structural and psychosocial factors to promote social connection and emotional well-being worldwide.

**Supplementary Information:**

The online version contains supplementary material available at https://doi.org/10.1007/s10654-026-01424-z.

## Introduction

Human beings are profoundly social. However, growing evidence shows that feelings of disconnection, reflected in loneliness arising from a gap between desired and actual social connection, are widespread and carry significant implications across the globe [1–5]. Loneliness has been linked to a broad array of psychological, emotional, and behavioral outcomes, including reduced well-being [5, 6] and increased risk for poor mental [5, 7, 8], physical health [9, 10], and mortality [11, 12]. It has therefore become a recognized public health concern, with several governments, including those of the United States, Japan, and South Korea, framing it as a “loneliness epidemic” [13–15]. In recent years, several high-income countries have established national loneliness strategies or appointed ministers for loneliness, reflecting growing recognition that social disconnection may threaten both individual and societal well-being [15].

Despite this growing attention, empirical research on loneliness remains limited in both scope and coverage [16]. Existing studies have primarily focused on older adults or other groups of interest [17, 18], country-level data [19, 20], a few high-income countries [21], meta studies [22, 23], or used global but non-representative samples [24]. This leaves major gaps in our understanding of how loneliness is distributed across different cultural, economic, and geopolitical contexts, its predictors, and how it relates to broader indicators of well-being. Addressing these questions is particularly urgent given global trends such as urbanization, migration, digitalization, and aging, all of which are likely reshaping patterns of social connection and isolation [25].

This study presents what we believe to be the first globally representative analysis of loneliness, its prevalence, predictors, and associations with several indicators of well-being. Drawing on > 218,000 individual-level responses from the 2023–2024 Gallup World Poll [26], spanning 148 countries, we pursue three primary objectives. Taken together, these objectives aim to offer a global perspective on the distribution and experience of loneliness and its potential associations with various factors associated with individual well-being [6–9]. In addition to examining current well-being, we also introduce a novel focus on individuals’ outlook for life, namely, their expected well-being in the future, which may provide insights into the forward-looking implications of loneliness.

First, we document the global prevalence of loneliness and examine how it varies across levels of economic development and geographic regions. While loneliness is increasingly recognized as a public health concern [13–15], most existing research has focused on high-income settings [17–21]. This study aims to provide the first consistent, globally representative evidence on loneliness, offering a broader perspective on where and for whom loneliness is most common, information that could help inform global health and development agendas [25].

Second, we identify key individual, household, and contextual predictors of loneliness and assess the heterogeneity in these associations across different dimensions, including economic development and world region. The choice of variables is guided by prior research on the determinants of loneliness [27–31] and by the measures available in the GWP dataset. This study seeks to strengthen the case for treating loneliness as a critical public health issue by providing a global analysis of its potential predictors across 148 countries, including many low- and middle-income contexts that have been underrepresented in prior research [21–24]. By simultaneously examining multiple dimensions of well-being, spanning subjective well-being, emotional experiences, and migration intentions, this paper builds on prior evidence focused primarily on high-income settings. Understanding these predictors may help clarify the extent to which loneliness reflects universal risk factors versus context-specific conditions, such as structural disadvantage, cultural norms, or social fragmentation. This distinction is important for informing policy and intervention design. If the predictors of loneliness are consistent across contexts, broad-based strategies may be effective; if they differ, responses may need to be tailored to specific cultural, social, or economic conditions. Building on existing evidence that highlights the health and social consequences of loneliness [5–12], findings from this study may contribute to future investments in social infrastructure, mental health services, and community-building initiatives aimed at reducing isolation and promoting psychosocial resilience globally.

Third, we analyze how loneliness relates to broader well-being outcomes, including both current and expected future life evaluations, as well as emotional states and migration intentions [5–8]. Understanding these associations is important for two key reasons. First, it allows us to assess the scope of loneliness not merely as an isolated emotional experience, but as a condition that may have measurable consequences for current well-being, life outlook, and behavior. If loneliness is strongly linked to lower subjective well-being, increased negative affect, and behavioral intentions such as emigration, it could signal a broader erosion of psychological and social functioning that may strain both individuals and communities. Second, recognizing loneliness as a potential driver of diminished well-being would support efforts to treat it as a critical public health issue, on par with other social determinants such as poverty, unemployment, or poor health. For policymakers, this highlights the potential value of investing in social infrastructure, mental health services, and community-building initiatives aimed at reducing isolation and fostering psychosocial resilience.

By offering a comprehensive, comparative account of loneliness across diverse countries and regions, this study aims to generate new insights into the social, structural, and emotional conditions associated with loneliness. In doing so, it seeks to contribute to both academic understanding and policy development, while underscoring the relevance of interventions that foster social connection and well-being. The findings may support the growing body of research on social behavior and mental health, while aligning with global efforts to address loneliness as a public health priority [13–16]. Furthermore, by contributing to the achievement of United Nations Sustainable Development Goal 3.4, promoting mental health and well-being for all, this research underscores the importance of global cooperation in addressing one of the most pressing social challenges.

## Methods

### Data set construction

In this study, we used data from two waves of the Gallup World Poll (GWP) conducted between 2023 and 2024, following the introduction of a new question on loneliness in 2023. The dataset comprised 218,048 individual survey responses across 148 countries (see Table S1). Importantly, these waves included different participants in each year, meaning the data were cross-sectional rather than longitudinal. The GWP employed nationally representative samples of adults aged 15 and older, covering approximately 99% of the global population [26]. In most countries, 1,000 individuals were surveyed, with larger samples in China (5,000) and India (3,000). Respondents were selected through a stratified random sampling method using the Kish grid. Interviews were conducted by telephone in countries with at least 80% coverage and face-to-face in others. While large-scale international surveys may have faced challenges such as translation inconsistencies or framing effects, Gallup applied stringent quality control procedures to minimize such issues and ensure data reliability [26].

### Key variable: loneliness

Measuring loneliness was difficult due to the latent nature of the construct: it could not be directly observed and had to be inferred from self-reported experiences. Various approaches existed for capturing loneliness, including direct versus indirect questioning, and single-item versus multi-item measures. In line with established survey practices, the Gallup World Poll employed a single, direct item to assess loneliness [32–34]. Our primary outcome variable was loneliness, measured through the question: “*Did you experience the following feelings during a lot of the day yesterday? How about loneliness?*” Respondents who answered “yes” were coded as experiencing loneliness.

This single-item direct measure offered several practical advantages [34]. Single-item measures were simple to administer, highly acceptable to participants, and directly targeted the subjective feeling of loneliness, which was the core of the construct. This made them especially useful in large-scale, cross-country surveys where brevity and clarity were essential [35]. Importantly, research had shown high correlations and strong agreement between single-item and multi-item, as well as between direct and indirect, measures of loneliness, supporting the validity of using a single, direct item [33, 36, 37], and the consistency of its association with various outcomes [37, 38]. This approach aligned with established practices in large-scale surveys and provided a consistent, comparable measure across diverse global contexts. In addition, the item’s temporal framing, focusing on whether the respondent felt lonely “a lot of the day yesterday”, reflected a state-based approach to loneliness, which was increasingly used in recent research [35, 39]. This literature highlighted that loneliness fluctuated meaningfully over short time frames and that state loneliness was a valid and important predictor of well-being, emotional experience, and health outcomes. This framing used by GWP aligned with its broader experiential well-being measures and enabled standardized, recall-reducing assessments of recent emotional experiences across countries and populations.

### Predictors of loneliness

Our analysis incorporated a comprehensive set of covariates capturing individual, household, and contextual characteristics that may have influenced loneliness. The selection of these variables was informed by prior research on the predictors of loneliness [27–31] and was constrained by data availability within the GWP. Demographic controls included age, gender (measured as a female-male binary indicator), and immigration status. Marital status was categorized into three groups: single; married or living with a partner; and separated, widowed, or divorced. Educational attainment was measured using categorical indicators for the highest level completed: elementary, secondary, or post-secondary education. A binary “children” variable indicated whether the respondent resided with at least one child (recoded from the number of children).

Household-level characteristics included the number of adults living in the household and annual per-capita household income. Income was reported in local currency and converted to international dollars using the World Bank’s purchasing power parity (PPP) private consumption conversion factor. To address nonresponse, income brackets were offered during data collection, and missing values were imputed using multiple imputation. Employment status was categorized as unemployed, out of the workforce, self-employed, or employed (full- or part-time). Urbanicity was captured through a binary variable distinguishing residents of urban areas (large cities or suburbs) from those in rural or small-town settings.

We also accounted for various psychosocial and contextual factors. Health status was measured by a binary variable indicating whether the respondent reported a health problem that prevented them from engaging in activities typical for their age group. Religiosity, a proxy for social and community engagement, was captured by whether religion was considered an important part of daily life. Social capital was assessed through responses to the question: “*If you were in trouble*,* do you have relatives or friends you can count on to help you whenever you need them?*” While often considered a measure of perceived social support, this item is widely used as an indicator of bonding social capital. Although it does not capture structural isolation, it reflects access to supportive ties, a key dimension of social capital. Importantly, this variable allowed us to distinguish social isolation (i.e., the objective lack of interactions) from loneliness (i.e., the subjective feeling about the gap between the level of social connection a person desires and what they actually experience), following [4, 5, 21]. Feelings of personal safety were measured by asking respondents whether they felt safe walking alone at night in the area where they lived. Finally, institutional trust was captured by a binary indicator of whether the respondent had confidence in the national government.

Subjective well-being was assessed using the 11-point Cantril Self-Anchoring Ladder, where respondents rated both their current life satisfaction and where they expected to stand in five years. The forward-looking version of this measure had been widely used in global well-being research as an indicator of optimism and perceived future quality of life [40, 41]. Prior studies had shown that expectations about future life evaluation reflected individuals’ broader sense of hope, agency, and life prospects, particularly in relation to their current economic, social, and health conditions. As such, the future-oriented question complemented assessments of present well-being by capturing individuals’ anticipated trajectories and outlook.

Relatedly, the GWP included a set of questions on emotional experiences, enabling us to explore whether loneliness was linked to specific emotions and, in turn, influenced well-being. We constructed binary indicators for whether respondents reported experiencing stress, anger, worry, pain, or enjoyment on the day prior to the survey. These emotional measures are the items consistently available across all GWP waves and countries; although most capture negatively valenced emotions, enjoyment provides a positively valenced counterpart. To capture international migration intentions, we used responses to the question: “*Ideally*,* if you had the opportunity*,* would you like to move permanently to another country*,* or would you prefer to continue living in this country?*” While intentions do not always lead to actual migration, prior research indicated that they were strong predictors of future migration behavior [42, 43].

Finally, countries in our sample were classified into three economic development tiers based on the World Bank’s gross national income (GNI) per capita thresholds [44]: low-income (≤$995), middle-income (>$996 and ≤$12,055), and high-income (>$12,056).

In line with prior theoretical and empirical work on loneliness [1–5, 27–31], we conceptually distinguished between predictors and outcomes of loneliness. Predictors included demographic, socioeconomic, and psychosocial characteristics (e.g., age, income, education, health, and social capital) that were typically understood as antecedent conditions or associations influencing the experience of loneliness. Outcomes included measures of subjective well-being, emotional states, and migration intentions, which were hypothesized to be affected by loneliness. We emphasized that, given the cross-sectional design of the Gallup World Poll, these relationships should be interpreted as associations rather than causal effects.

### Statistical methods

Descriptive and regression analyses were employed to examine the relationships between the previously identified predictors and loneliness. Descriptive analyses incorporated Gallup’s sampling weights to ensure national representativeness and included summary statistics and Pearson’s correlation coefficients to characterize bivariate associations between loneliness and various correlates. To identify predictors of loneliness, we estimated multivariate logistic regression model controlling for a comprehensive set of predictors and fixed effects. For individual *i* in country *j* and year *t*, the model was specified as:

$$\:{Lonely}_{i,j,t}=\alpha\:+\beta\:{X}_{i,j,t}+{\vartheta\:}_{j}+{\varphi\:}_{t}+{\epsilon\:}_{i,j,t},$$

where $\:{X}_{i,j,t}$ is the vector of covariates, which included age, gender, immigration status, marital status, educational attainment, employment status, income, number of adults in the household, presence of children, urban versus rural residence, self-reported health limitations, religiosity, social capital, feelings of personal safety, and confidence in the government. Furthermore, $\:{\vartheta\:}_{j}$ and $\:{\varphi\:}_{t}$ represent country and year fixed effects, respectively. We included these to account for unobserved heterogeneity that could bias our results. Country fixed effects control for stable, time-invariant differences across countries (e.g., cultural norms, public policies, and institutional frameworks) that may influence how individuals experience and report loneliness. This is particularly important in cross-national studies of loneliness, as cultural differences can shape both the prevalence and interpretation of loneliness in significant ways. Year fixed effects capture time-specific shocks and trends (e.g., global economic conditions, international events, or changes in survey context) that affect all countries in a given year. Together, these fixed effects helped isolate the variation of interest by holding constant the influence of stable country characteristics and temporal effects, thereby improving the validity of cross-national comparisons.

To examine how loneliness relates to well-being outcomes, including current and future life evaluations, emotional experiences (e.g., stress, worry, enjoyment), and migration intentions, we estimated additional multivariate models using ordinary least squares for continuous outcomes and logistic regression for binary outcomes, using the same model as above including the same covariates and fixed effects. Standard errors were clustered at the country level to account for within-group correlation for all models. Following standard practices for GWP data, survey weights were not applied in regressions.

For all logistic regressions, we report average marginal effects (AMEs) rather than odds ratios, as AMEs provide directly interpretable estimates of the average change in outcome probability associated with a one-unit change in the predictor, facilitate comparison across models and groups, and avoid the non-collapsibility problem inherent to odds ratios; an important advantage given the non-rare prevalence of loneliness in many populations.

Finally, missing data were addressed using listwise deletion (complete-case analysis) in the main regression models to maintain consistency and comparability across countries, following standard practice in analyses of GWP data. To evaluate the potential impact of missingness, we conducted sensitivity analyses employing multiple imputation and full information maximum likelihood estimation. These supplementary analyses yielded results consistent with the complete-case models, indicating that missing data are unlikely to substantially bias our findings. All analyses were conducted using Stata/SE 18.

## Results

### Sample characteristics

Table 1 presents summary statistics for the full sample, providing an overview of key demographic, socio-economic, and psychosocial characteristics. We found that 21.3% ± 40.9% of individuals reported feeling lonely, highlighting the global relevance of loneliness as an emotional and social issue. However, substantial variation was observed across levels of economic development and regions (Table S2). Loneliness was most prevalent in low-income countries, where 31.3% of respondents reported experiencing it, compared to 23.1% in middle-income and 15.7% in high-income countries. Regional disparities were also pronounced: the prevalence of loneliness was highest in Sub-Saharan Africa (SSA; 30%), followed by the Middle East and North Africa (MENA; 24.7%) and the Americas (20.1%), while it was lowest in Europe (16.2%) and Asia (18.6%).

**Fig. 1:**
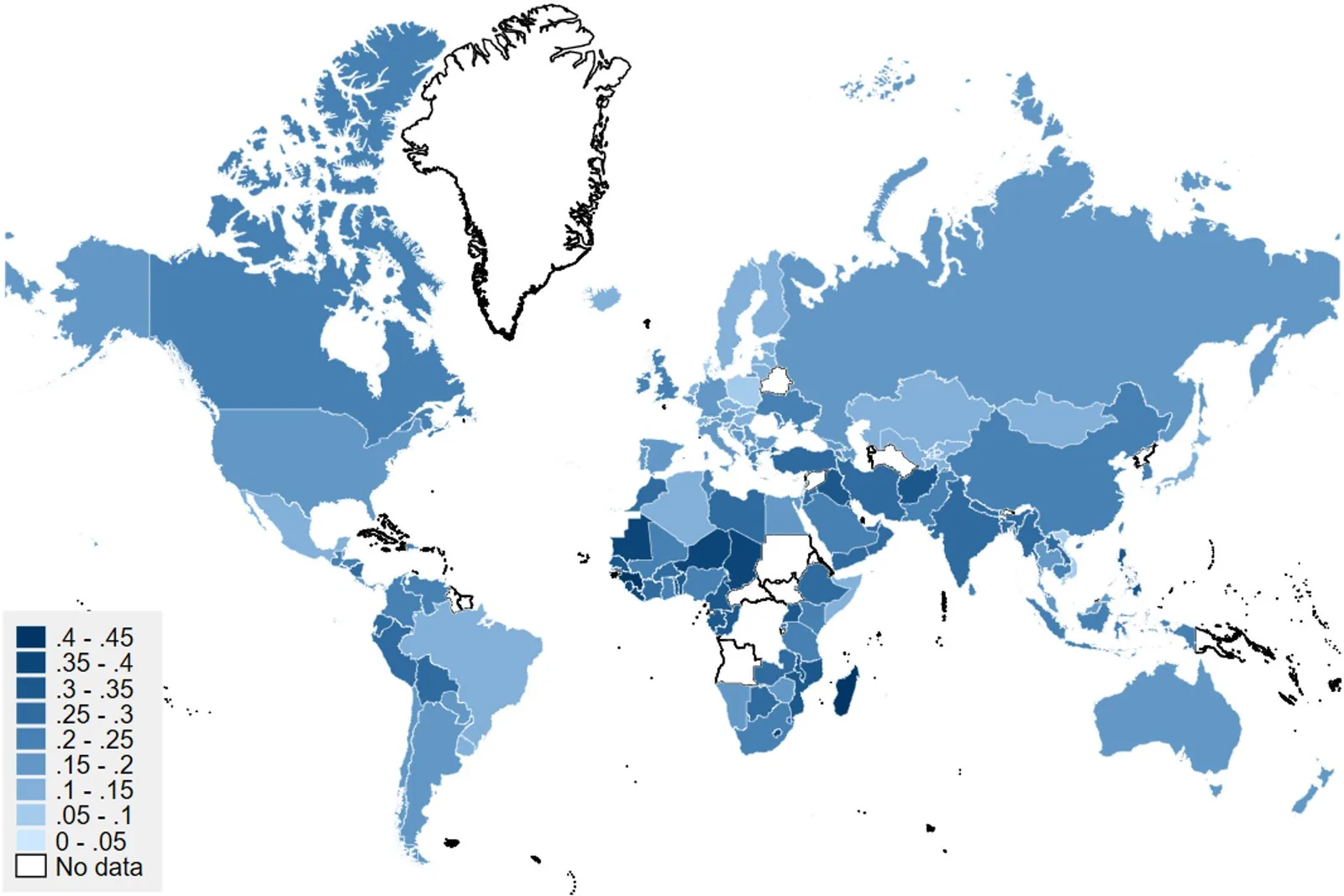
Global prevalence of loneliness, 2023–2024. Figure shows the country-level prevalence rate of loneliness. Data are from individual-level Gallup World Poll data for 2023–2024 and 148 countries. Sample weights were applied

These differences are visualized in Fig. 1, which presents a global map of loneliness prevalence, offering a representation of the underlying geographic disparities.

Beyond loneliness, the sample was broadly diverse in terms of demographic, economic, and psychosocial characteristics. The average age was 41.5 years, with an even gender distribution. The majority of respondents had completed secondary education, and just over half reported living with children. About 27% reported a health problem that limited daily activities, and 68% considered religion important in daily life. Trust in the government was reported by 54% of respondents, while 80% indicated that they had high social capital, and 42% lived in an urban area. In terms of emotional states, nearly 37% reported stress, 40% worry, 34% pain, and 20% anger, while 72% reported experiencing enjoyment. The average scores for current and future subjective well-being were 5.76 and 7.18, respectively, and roughly 26% of respondents expressed an intention to migrate permanently to another country. Mean income was $10,251±$46,597, with most respondents being part- or full-time employed.

Loneliness was consistently negatively correlated with both current and future SWB across all income and regional groups (Tables S3 and S4). Correlations were strongest in high-income countries and in Europe, where loneliness showed the largest negative association with life evaluations. Conversely, loneliness was positively correlated with negative emotions such as stress, anger, worry, and pain, with particularly strong correlations observed in low- and middle-income countries, as well as in Asia and MENA. Enjoyment showed a moderate negative correlation with loneliness across all groups.

**Table 1:** Sample characteristics

Variable	Mean	Std. dev.	Min	Max	Obs.
Lonely	0.213	0.409	0	1	218,048
Current SWB	5.757	2.534	0	10	216,591
Future SWB	7.183	2.481	0	10	204,309
Income	10,251.18	46,597.3	0	7,186,563	218,048
Age	41.541	17.987	15	99	218,048
Male	0.491	0.500	0	1	218,048
Religiosity	0.677	0.468	0	1	218,048
Education					
Elementary	0.337		0	1	218,048
Secondary	0.507		0	1	218,048
Post-secondary	0.156		0	1	218,048
Marital status					
Single	0.332		0	1	218,048
Married/partner	0.538		0	1	218,048
Separated/divorced/widowed	0.130		0	1	218,048
Adults	4.207	2.862	1	20	218,048
Children	0.506	0.500	0	1	218,048
Health problem	0.271	0.444	0	1	218,048
Immigrant	0.062	0.240	0	1	218,048
Urban	0.422	0.494	0	1	218,048
Social capital	0.804	0.397	0	1	218,048
Safe	0.665	0.472	0	1	218,048
Trust	0.543	0.498	0	1	218,048
Employment status					
Unemployed	0.064		0	1	218,048
Out-of-workforce	0.346		0	1	218,048
Part-time employed	0.161		0	1	218,048
Full-time employed	0.429		0	1	218,048
Stress	0.369	0.483	0	1	217,445
Anger	0.197	0.398	0	1	217,580
Worry	0.403	0.491	0	1	217,609
Pain	0.343	0.475	0	1	217,790
Enjoyment	0.718	0.450	0	1	216,928
Migration intention	0.258	0.438	0	1	215,508

Data are from the Gallup World Poll (2023–2024). Sample weights were applied. Categorical and binary variables are presented as proportions (percentages) of the total sample within each category. Continuous variables, such as age and income, are reported as means with standard deviations

Finally, to investigate whether loneliness has been increasing over time, a concern often framed as a potential “loneliness epidemic” [13–15], we analyzed year-to-year changes in prevalence across countries. Across the 113 countries with data available in both years, the average change in reported loneliness was − 0.3pp (median − 0.7pp). Most countries (61%; see Figure S1) experienced a decline in the prevalence of loneliness. However, there was no evidence to suggest that the average change in loneliness prevalence across countries significantly differed from zero. When disaggregated by World Bank income groups, patterns varied: loneliness prevalence increased by 3.4pp in low-income countries (*p*<.01), remained stable in middle-income countries (−0.3pp; *p*=.4), and decreased in high-income countries by 1pp (*p*<.01).

### Predictors of loneliness

Table 2 presents our results examining the associations between individual and contextual characteristics and the probability of reporting loneliness.

A range of demographic, socioeconomic, and psychosocial factors were significantly associated with loneliness in the full sample. Income had a non-linear relationship with loneliness, but across most of its range, higher income was associated with lower levels of loneliness. Age showed a concave relationship with loneliness: initially increasing with age but declining from 50 years onwards. Male respondents were 0.9% points (pp) more likely to report loneliness than female respondents (95% CI, 0.4–1.4pp; *p*<.01), and religiosity was positively associated with loneliness, with individuals who considered religion important being 2.4pp more likely to report loneliness (95% CI, 1.8–2.9pp; *p*<.01).

Higher levels of education were associated with a lower likelihood of reporting loneliness. Respondents with secondary education were 3.2pp less likely to report loneliness than those with elementary education (95% CI, −4.3 to −2.2pp; *p*<.01) while respondents with post-secondary education were 5.3pp less likely to report loneliness than those with elementary education (95% CI, −6.4 to −4.3pp; *p*<.01). Marital status also showed strong associations: compared to singles, individuals who were married or living with a partner were 9.1pp less likely to report loneliness (95% CI, −10.3 to −8pp; *p*<.01), whereas those who were separated, divorced, or widowed were 8.9pp more likely to report loneliness (95% CI, 7.5–10.4pp; *p*<.01).

Household composition mattered. A larger number of adults in the household and the presence of children were both associated with lower probabilities of loneliness. For each additional adult in the household, the likelihood of loneliness decreased by 1pp (95% CI, −1.2 to −0.7pp; *p*<.01). The presence of children in the household was associated with a 1.5pp lower likelihood of loneliness (95% CI, −2.2 to −0.8pp; *p*<.01). Respondents who reported having a health problem were 10.4pp more likely to report loneliness (95% CI, 9.7–11.1pp; *p*<.01). Immigrant respondents were 2.8pp more likely to report loneliness than non-immigrants (95% CI, 1.8–3.8pp; *p*<.01) and living in an urban area compared to a rural area was associated with a 0.8pp lower likelihood of loneliness (95% CI, −1.4 to −0.3pp; *p*<.01).

Social and contextual factors also showed strong associations with loneliness. Individuals with high social capital were 8pp less likely to report loneliness (95% CI, −9.0 to −6.9pp; *p*<.01). Similarly, those who felt safe were 3pp less likely to report loneliness (95% CI, −3.6 to −2.4pp; *p*<.01), and those who trusted the government were 1.8pp less likely to report loneliness (95% CI, −2.4 to −1.2pp; *p*<.01). Compared to unemployed individuals, those out of the workforce were 6pp less likely to report loneliness (95% CI, −7 to −5pp; *p*<.01), part-time employed 4pp less likely (95% CI, −5.2 to −2.8pp; *p*<.01), and full-time employed 6.6pp less likely (95% CI, −7.7 to −5.6pp; *p*<.01).

**Table 2:** Predictors of loneliness

	Full sample	Low-income	Middle-income	High-income
Log(income)	0.003	−0.009	0.004	0.007^**^
	[−0.001,0.007]	[−0.028,0.011]	[−0.002,0.010]	[0.001,0.013]
Log(income) squared	−0.001^***^	−0.000	−0.001^***^	−0.001^***^
	[−0.001,−0.001]	[−0.002,0.001]	[−0.002,−0.001]	[−0.002,−0.001]
Age	0.003^***^	−0.000	0.004^***^	0.001
	[0.002,0.004]	[−0.003,0.003]	[0.003,0.006]	[−0.001,0.002]
Age–squared	−0.000^***^	0.000	−0.000^***^	−0.000^**^
	[−0.000,−0.000]	[−0.000,0.000]	[−0.000,−0.000]	[−0.000,−0.000]
Male	0.009^***^	0.001	0.002	0.020^***^
	[0.004,0.014]	[−0.012,0.015]	[−0.005,0.009]	[0.012,0.027]
Religion	0.024^***^	0.016	0.017^***^	0.027^***^
	[0.018,0.029]	[−0.025,0.057]	[0.008,0.027]	[0.020,0.033]
Educational status				
Secondary	−0.032^***^	0.010	−0.037^***^	−0.034^***^
	[−0.043,−0.022]	[−0.008,0.028]	[−0.049,−0.024]	[−0.047,−0.020]
Post-secondary	−0.053^***^	0.006	−0.065^***^	−0.042^***^
	[−0.064,−0.043]	[−0.041,0.052]	[−0.080,−0.049]	[−0.056,−0.029]
Marital status				
Married/partner	−0.091^***^	−0.037^***^	−0.090^***^	−0.096^***^
	[−0.103,−0.080]	[−0.062,−0.012]	[−0.107,−0.074]	[−0.109,−0.083]
Separated/divorced/widowed	0.089^***^	0.089^***^	0.101^***^	0.068^***^
	[0.075,0.104]	[0.057,0.121]	[0.081,0.122]	[0.046,0.090]
Household size	−0.010^***^	−0.004^***^	−0.012^***^	−0.015^***^
	[−0.012,−0.007]	[−0.006,−0.001]	[−0.016,−0.008]	[−0.019,−0.011]
Children	−0.015^***^	−0.016	−0.018^***^	0.003
	[−0.022,−0.008]	[−0.042,0.009]	[−0.030,−0.007]	[−0.006,0.013]
Health problem	0.104^***^	0.114^***^	0.107^***^	0.084^***^
	[0.097,0.111]	[0.094,0.135]	[0.099,0.116]	[0.074,0.094]
Immigrant	0.028^***^	0.003	0.023^***^	0.023^***^
	[0.018,0.038]	[−0.028,0.034]	[0.006,0.040]	[0.011,0.035]
Urban	−0.008^***^	0.002	−0.013^***^	−0.006^*^
	[−0.014,−0.003]	[−0.018,0.023]	[−0.021,−0.004]	[−0.013,0.001]
Social capital	−0.080^***^	−0.053^***^	−0.074^***^	−0.115^***^
	[−0.090,−0.069]	[−0.073,−0.034]	[−0.086,−0.061]	[−0.128,−0.101]
Safe	−0.030^***^	−0.004	−0.030^***^	−0.039^***^
	[−0.036,−0.024]	[−0.034,0.025]	[−0.037,−0.024]	[−0.047,−0.031]
Confidence government	−0.018^***^	0.016	−0.018^***^	−0.026^***^
	[−0.024,−0.012]	[−0.008,0.040]	[−0.025,−0.010]	[−0.032,−0.019]
Employment status				
Out-of-workforce	−0.060^***^	−0.089^***^	−0.073^***^	−0.032^***^
	[−0.070,−0.050]	[−0.114,−0.065]	[−0.086,−0.059]	[−0.046,−0.017]
Part-time employed	−0.040^***^	−0.032^**^	−0.049^***^	−0.033^***^
	[−0.052,−0.028]	[−0.061,−0.004]	[−0.066,−0.031]	[−0.052,−0.013]
Full-time employed	−0.066^***^	−0.059^***^	−0.078^***^	−0.049^***^
	[−0.077,−0.056]	[−0.082,−0.037]	[−0.094,−0.062]	[−0.064,−0.034]
Obs.	218,048	21,748	116,895	78,002
Pseudo R^2^	0.10	0.04	0.10	0.10

Table shows average marginal effects from logit regressions. All regressions included country and year fixed effects. Omitted category for education is elementary, marital status is single, and employment is unemployed. World Bank’s gross national income (GNI) per capita thresholds used for classification: low-income (≤$995), middle-income (>$996 and ≤$12,055), and high-income (>$12,056). Clustered-standard errors at the country-level were used and 95% Confidence intervals in parentheses. Significance levels: *: *p*<.1, **: *p*<.05, ***: *p*<.01

When the analysis was disaggregated by economic development, several patterns emerged. Income and age were significantly associated with loneliness in middle- and high-income countries, but not in low-income countries. Gender differences in loneliness were statistically significant only in high-income countries, where men were more likely to report loneliness. The positive association between religiosity and loneliness was observed in middle- and high-income countries, but not in low-income countries. Higher education levels were associated with lower loneliness in middle- and high-income countries, with no significant association in low-income countries. Being married or partnered was associated with lower loneliness in all income groups, while being separated, divorced, or widowed was associated with higher loneliness across the board. Larger household size was associated with lower loneliness in all country groups, with the strongest association in high-income settings. The presence of children was significantly associated with lower loneliness only in middle-income countries. Health problems were consistently and significantly associated with higher loneliness across all groups. Immigrant status was positively associated with loneliness in middle- and high-income countries, but not in low-income contexts. Urban residence was negatively associated with loneliness in middle- and high-income countries. Social capital was associated with lower loneliness across all income groups, with the strongest association observed in high-income countries. Feeling safe and trust in the government were significantly associated with lower loneliness in middle- and high-income countries only. Employment status showed consistent associations across income groups. In all groups, those who were employed or out of the workforce were less likely to report loneliness than the unemployed, though the magnitude of the association varied.

Finally, we examined whether the predictors of loneliness varied by region (Table S5). While many associations held across contexts, key differences emerged. Income showed a significant non-linear association with loneliness in Europe and the Americas, but not in Asia or SSA. Age was more strongly linked to loneliness in Asia, the Americas, and MENA, while gender was only significant in Europe and SSA. Education was consistently negatively associated in all regions except SSA. Being married or partnered was associated with lower loneliness everywhere, but the strength of the association varied. Health problems, low social capital, and unemployment were consistently linked to higher loneliness across all regions. Some factors, like urban residence and feeling safe, were more salient in Europe and MENA.

### Associations between loneliness and well-being

Beyond identifying the predictors of loneliness, we examined its relationship with subjective well-being, a widely recognized indicator of quality of life and a growing focus in global development and public health agendas.

In Table 3, across the full sample, loneliness was significantly and negatively associated with both current and expected future SWB. Respondents who reported feeling lonely rated their current SWB 0.48 points lower on the 11-point Cantril ladder than those who did not report loneliness (95% CI, −0.54 to −0.42; *p*<.01). The association between loneliness and future SWB was also negative, though smaller in magnitude: lonely individuals rated their expected future SWB 0.1 points lower than non-lonely individuals (95% CI, −0.13 to −0.07; *p*<.01).

**Table 3:** Associations between loneliness and subjective well-being

	Current SWB			
	Full sample	Low-income	Middle-income	High-income
Lonely	−0.480^***^	−0.202^**^	−0.446^***^	−0.654^***^
	[−0.539,−0.420]	[−0.360,−0.044]	[−0.523,−0.368]	[−0.729,−0.579]
Obs.	216,591	21,534	115,844	77,817
Adj. R^2^	0.25	0.09	0.18	0.21

	Future SWB			
	Full sample	Low-income	Middle-income	High-income
Lonely	−0.100^***^	−0.025	−0.094^***^	−0.063^***^
	[−0.129,−0.072]	[−0.111,0.060]	[−0.136,−0.053]	[−0.109,−0.016]
Obs.	203,739	20,569	106,675	75,225
Adj. R^2^	0.38	0.22	0.36	0.52

All regressions included controls (shown in the appendix; Table S6 and S7) and country and year fixed effects. World Bank’s gross national income (GNI) per capita thresholds used for classification: low-income (≤$995), middle-income (>$996 and ≤$12,055), and high-income (>$12,056). Standard errors were clustered at the country level and 95% Confidence intervals in parentheses. Significance levels: *: *p*<.1, **: *p*<.05, ***: *p*<.01

This pattern held when we disaggregated the sample by countries’ economic development: the associations became stronger in more economically developed countries. In high-income countries, loneliness was associated with a 0.65-point lower current SWB (95% CI, −0.73 to −0.58; *p*<.01) and a 0.06-point lower future SWB (95% CI, −0.11 to −0.02; *p*<.01). In middle-income countries, lonely respondents rated their current SWB 0.45 points lower (95% CI, −0.52 to −0.37; *p*<.01) and their future SWB 0.09 points lower (95% CI, −0.14 to −0.05; *p*<.01). In low-income countries, loneliness was associated with a 0.2-point lower current SWB (95% CI, −0.36 to −0.04; *p*<.05), while the association with future SWB was not statistically significant.

We also examined whether the association between loneliness and SWB varied across regions (Table S8, S9). Loneliness was negatively associated with both current and future SWB in all regions, but the strength of the relationship differed. For current SWB, the association was strongest in MENA (−0.68; 95% CI, −1.11 to −0.26pp; *p*<.01), Europe (−0.64; 95% CI, −0.72 to −0.55pp; *p*<.01), the Americas (−0.56; 95% CI, −0.67 to −0.45pp; *p*<.01), and Asia (−0.52; 95% CI, −0.63 to −0.4pp; *p*<.01) and notably weaker in SSA (−0.23; 95% CI, −0.33 to −0.13pp; *p*<.01). For future SWB, the coefficients were smaller, but still negative, with the largest associations in the Americas (−0.1; 95% CI, −0.18 to −0.03pp; *p*<.05) and Europe (−0.05; 95% CI, −0.1 to −0.003pp; *p*<.05). In Asia, MENA, and SSA the associations were not significant.

### Associations between loneliness, emotions, and behavioral intentions

To further explore how loneliness related to broader well-being outcomes, we examined its association with several emotional states. Table 4 shows that lonely individuals were 23pp more likely to report feeling stressed (95% CI, 21.5–24.6pp; *p*<.01), 17.4pp more likely to report anger (95% CI, 16.2–18.7pp; *p*<.01), 24.6pp more likely to report worry (95% CI, 23.2–25.9pp; *p*<.01), and 12.9pp more likely to report physical pain (95% CI, 12–13.7pp; *p*<.01) than non-lonely individuals. Conversely, loneliness was associated with a 12.2pp lower likelihood of reporting enjoyment (95% CI, −13.5 to −10.8pp; *p*<.01).

We also assessed the association between loneliness and migration intentions, a forward-looking behavioural outcome often linked to social disconnection and weakened social networks [45]. Lonely individuals were 4.5pp more likely to express a desire to move permanently to another country (95% CI, 4–5.1pp; *p*<.01) than those who did not report feeling lonely.

Finally, Figures S2 and S3 present the heterogeneity by economic development and region. Overall, the strength of the association between loneliness and emotional experiences or behaviors weakened as economic development increased. Furthermore, we generally found smaller associations in Europe and SSA compared to MENA and the Americas.

**Table 4:** Associations between loneliness and emotional/behavioral outcomes

	Stress	Anger	Worry	Pain	Enjoyment	Migration intention
Lonely	0.230^***^	0.174^***^	0.246^***^	0.129^***^	−0.122^***^	0.045^***^
	[0.215,0.246]	[0.162,0.187]	[0.232,0.259]	[0.120,0.137]	[−0.135,−0.108]	[0.040,0.051]
Obs.	217,445	217,580	217,609	217,790	216,928	215,508
Pseudo R^2^	0.11	0.10	0.11	0.14	0.10	0.18

Table presents average marginal effects from logit regressions. All regressions include controls (shown in the Table S10) and country and year fixed effects. Standard errors clustered at the country level and 95% Confidence intervals in parentheses. Significance levels: *: *p*<.1, **: *p*<.05, ***: *p*<.01

## Discussion

This study presented the first comprehensive, globally representative assessment of loneliness, examining its predictors, its associations with various well-being indicators, and how these patterns varied across geographic regions and levels of economic development.

Our analysis revealed that loneliness was a widespread global experience [22], affecting more than one in five individuals, with substantial variation across geographic regions and levels of economic development. Prevalence was highest in low-income countries and in regions marked by poverty, instability, or limited social infrastructure, such as SSA, MENA, and parts of the Americas. These disparities likely reflected both structural and cultural factors, including limited access to mental health care, greater social fragmentation due to conflict or economic hardship, and differing norms around emotional expression and social support [24]. However, loneliness was far from absent in high-income countries, where social services tended to be more robust and average well-being was typically higher [46]. Notably, in these wealthier contexts, loneliness was most strongly associated with reduced well-being, suggesting that in settings where basic needs were more consistently met and social expectations were higher, the experience of loneliness may have been more psychologically dissonant and thus more strongly linked to individuals’ overall well-being [5].

By examining a wide range of predictors, this study identified key factors associated with loneliness. Among these, social capital, education, household composition, employment, and health status emerged as the most consistent and influential associations [27–31]. Not surprisingly, social capital stood out as the strongest and most universal factor linked to lower loneliness: individuals with supportive networks and high levels of trust were significantly less likely to report loneliness across all income groups and regions. This supported longstanding sociological and epidemiological theories that emphasized the buffering role of social connections against emotional distress [27]. Other predictors, however, showed more context-specific patterns. Education, for example, was negatively associated with loneliness in high- and middle-income countries but showed no such association in low-income countries or Sub-Saharan Africa, suggesting that in settings where material deprivation remained high, education alone may not have been sufficient to counter broader forms of social disconnection [27, 28, 31]. Similarly, while health limitations, smaller households, and unemployment were generally positively associated with loneliness, the strength and significance of these associations varied by region and level of development. For instance, health limitations were strongly and consistently associated with greater loneliness across all regions, with particularly pronounced effects in middle- and high-income countries. This may have reflected higher expectations for active social participation and healthy aging in these contexts. In low-income settings, although the relationship remained significant, its relative impact may have been attenuated by other pressing socioeconomic hardships. Household size was more strongly linked to lower loneliness in Europe, Asia, and the Americas, consistent with research highlighting the protective role of co-residence and extended family structures, particularly in more collectivist cultures [24, 49]. The relationship between employment and loneliness also varied: being employed or being out of the workforce was associated with lower loneliness across all income groups, but the effect was strongest in middle- and low-income countries, where employment may have carried greater social and psychological significance. These variations underscored the importance of local cultural norms and economic conditions when interpreting predictors of loneliness [24, 28, 30].

Interestingly, although the prevalence of loneliness was lowest in high-income countries, its negative associations with well-being were strongest. Our analysis revealed consistently negative relationships between loneliness and both current and future SWB across regions. The relationship was strongest for current SWB, particularly in MENA, Europe, and the Americas, suggesting that loneliness was more strongly linked to lower current well-being in these regions [5–8]. The association with future well-being was more modest, likely because future outlooks were influenced by a broader set of aspirations, uncertainties, and external hopes [47]. Regional variation further underscored how cultural and contextual factors may have shaped these relationships. These findings aligned with research showing that loneliness was a key correlate of life dissatisfaction, even after controlling for objective socioeconomic factors [5–9].

Relatedly, loneliness was significantly associated with emotional burden [5, 8, 48]. Lonely individuals were significantly more likely to report negative emotional states and less likely to experience enjoyment. These patterns were stronger in less developed and more collectivist regions, such as MENA and Asia, where social connectedness played a central cultural role [24, 49]. The relationship between loneliness and migration intentions added a behavioral dimension to this emotional toll, suggesting that loneliness may not only have shaped internal states but also related to behavioral motivations [5, 48].

These findings supported theoretical frameworks that conceptualized loneliness as a form of social pain with wide-ranging consequences [1–5]. When individuals felt disconnected from meaningful relationships, they experienced psychological distress and may have withdrawn from their communities or considered substantial life changes, such as migration, to improve their social environment [5, 42].

Our results also aligned with Social Identity Theory [49], which emphasized that individuals derived well-being and self-concept from group membership and belonging. The stronger negative association between loneliness and well-being in high-income countries suggested that loneliness may have been especially difficult in settings where individualism and high social expectations prevailed. In these contexts, loneliness could have signaled exclusion from identity-relevant groups, which may have intensified its psychological toll [5, 8]. In contrast, in more collectivist societies, such as parts of MENA and Asia, where group affiliation was central to identity, loneliness may have reflected a deeper rupture in perceived belonging [24, 48]. This may have helped explain both the emotional burden of loneliness and its link to migration intentions, as individuals sought environments where their social identity could be restored [42, 45].

The association between social capital and reduced loneliness further supported theories highlighting the importance of trust, reciprocity, and social networks [50]. Supportive relationships emerged as one of the most consistent predictors of lower loneliness across all contexts. Disaggregating by type, bonding capital, seen in marriage, having children, or living in large households, may have provided close emotional support. Bridging capital, linked to education and urban living, was associated with broader, more diverse ties. Linking capital may have been limited by poor health or immigrant status, which could have restricted access to institutions and social participation. Additionally, trust and feelings of safety were negatively associated with loneliness, suggesting that secure environments may have supported greater engagement. These findings illustrated how different forms of social capital were related to loneliness globally [5, 8, 50].

Given ongoing debate about the lasting social consequences of COVID-19, we also explored whether perceived pandemic impacts were associated with loneliness [23, 51]. As an exploratory analysis, we examined whether self-reported impacts of the COVID-19 pandemic were associated with loneliness, using data from the 2023 wave (*N* = 108,963) of the GWP. Compared to respondents who reported that their lives were not at all affected by the pandemic, those who reported that their lives were affected “a lot” were 3.5pp more likely to report feeling lonely (95% CI, 2.7–4.4pp; *p*<.01). No statistically significant difference was found for those who reported only “some” impact.

Despite the notable strengths of our study, most notably its unprecedented global coverage [15], the use of a large and heterogeneous sample, and a rigorous analysis of both individual- and context-level predictors of loneliness, several limitations warranted consideration. First, the cross-sectional design limited our ability to draw causal conclusions. While we reported associations between loneliness and several indicators of well-being, including health status and life evaluation, it was important to emphasize the possibility of bidirectional relationships. For example, loneliness may have negatively impacted health and life satisfaction, but poor health or low life satisfaction may also have increased vulnerability to loneliness. Future research using longitudinal designs was needed to better understand the directionality and potential feedback loops between these factors. Second, the reliance on self-reported data introduced the possibility of response and reporting biases [32, 33]. Third, our measure of loneliness was based on a single survey item. While this approach was common in large-scale, cross-national research for reasons of feasibility and comparability, it may have lacked the nuance of multidimensional loneliness scales that distinguished between emotional, social, and existential forms of loneliness [33–38]. Furthermore, the use of a single-item measure asking about loneliness “yesterday” may have reflected short-term affect more than chronic loneliness. While prior research [e.g., 52] found moderate correlations between daily and global loneliness measures, this item may have captured transient rather than persistent social disconnection.

Given its consistent associations with well-being indicators, loneliness should be considered a global public health concern [13–15]. While our cross-sectional findings could not establish causality, they highlighted the potential value of both universal and context-sensitive strategies to address loneliness [53–55]. At the macro level, policies that aim to foster social capital, such as community programs, inclusive public spaces, and local engagement initiatives, may help reduce experiences of social disconnection. In low- and middle-income countries, addressing barriers such as mental health stigma [56] may support help-seeking and strengthen social support systems. Context-specific responses are particularly relevant in regions with high reported loneliness. Addressing broader structural factors like poverty, displacement, and social fragmentation through investments in social protection, employment, and education could contribute to both economic and social resilience. Urban design may also play a role; environments that promote inclusion, mobility, and informal social interaction might help mitigate isolation and foster connection [57]. Finally, governments and international organizations could consider incorporating loneliness indicators into national well-being and development frameworks. Routine monitoring may support more responsive, evidence-informed policymaking aimed at improving social and emotional well-being.

## Supplementary Information

Below is the link to the electronic supplementary material.

Supplementary file1 (DOCX 21 kb)
