# Supplementary material for: Loneliness around the world: patterns, predictors, and well-being implications
Source: Eur J Epidemiol. 2026 Jun 26;41(8):1005–17. doi: 10.1007/s10654-026-01424-z (PMC13612629; doi:10.1007/s10654-026-01424-z)
Supplement: Supplementary file 1 [file 10654_2026_1424_MOESM1_ESM.docx]

**Supplementary Material**

Table S1: List of Countries

| Afghanistan | Croatia | Israel | Mozambique | Slovenia |
| --- | --- | --- | --- | --- |
| Albania | Cyprus | Italy | Myanmar | Somalia |
| Algeria | Czech Republic | Ivory Coast | Namibia | South Africa |
| Argentina | Denmark | Japan | Nepal | South Korea |
| Armenia | Dominican Republic | Jordan | Netherlands | Spain |
| Australia | Ecuador | Kazakhstan | New Zealand | Sri Lanka |
| Austria | Egypt | Kenya | Nicaragua | Sweden |
| Azerbaijan | El Salvador | Kosovo | Niger | Switzerland |
| Bahrain | Estonia | Kuwait | Nigeria | Taiwan |
| Bangladesh | Eswatini | Kyrgyzstan | North Macedonia | Tajikistan |
| Belgium | Ethiopia | Laos | Northern Cyprus | Tanzania |
| Belize | Finland | Latvia | Norway | Thailand |
| Benin | France | Lebanon | Oman | Togo |
| Bolivia | Gabon | Lesotho | Pakistan | Trinidad and Tobago |
| Bosnia Herzegovina | Gambia | Liberia | Palestine | Tunisia |
| Botswana | Georgia | Libya | Panama | Turkey |
| Brazil | Germany | Lithuania | Paraguay | Uganda |
| Bulgaria | Ghana | Luxembourg | Peru | Ukraine |
| Burkina Faso | Greece | Madagascar | Philippines | United Arab Emirates |
| Cambodia | Guatemala | Malawi | Poland | United Kingdom |
| Cameroon | Guinea | Malaysia | Portugal | United States |
| Canada | Honduras | Mali | Romania | Uruguay |
| Chad | Hong Kong | Malta | Russia | Uzbekistan |
| Chile | Hungary | Mauritania | Rwanda | Venezuela |
| China | Iceland | Mauritius | Saudi Arabia | Vietnam |
| Colombia | India | Mexico | Senegal | Yemen |
| Comoros | Indonesia | Moldova | Serbia | Zambia |
| Congo Brazzaville | Iran | Mongolia | Sierra Leone | Zimbabwe |
| Congo Kinshasa | Iraq | Montenegro | Singapore |  |
| Costa Rica | Ireland | Morocco | Slovakia |  |

Table S2: Prevalence of Loneliness by Economic Development and Region

| **Panel A** | Full Sample |  | Low | Middle | High | Wald χ^2^ |
| --- | --- | --- | --- | --- | --- | --- |
| Lonely | 0.213 (0.409) |  | 0.313 (0.464) | 0.231 (0.421) | 0.157 (0.364) | 2,992.89*** |
| Obs. | 218,048 |  | 21,748 | 116,895 | 78,002 |  |
|  |  |  |  |  |  |  |
| **Panel B** | Europe | Asia | Americas | MENA | SSA |  |
| Lonely | 0.162 (0.368) | 0.186 (0.389) | 0.201 (0.401) | 0.247 (0.431) | 0.300 (0.458) | 3,416.50*** |
| Obs. | 62,213 | 55,960 | 36,178 | 9,844 | 53,853 |  |

Notes: Mean values of loneliness are reported with standard deviations in parentheses. Data are from the Gallup World Poll (2023-2024), and survey weights are applied. The Wald χ² test (assuming unequal variances) assesses differences in prevalence across groups, based on a test for equality of means.

Table S3: Correlations of Loneliness with Well-Being and Emotional States

| Correlation of lonely with… | Full Sample | Low | Middle | High |
| --- | --- | --- | --- | --- |
| SWB | -0.177 | -0.069 | -0.149 | -0.202 |
| Future SWB | -0.124 | -0.055 | -0.122 | -0.153 |
| Stress | 0.233 | 0.264 | 0.266 | 0.171 |
| Anger | 0.236 | 0.257 | 0.252 | 0.164 |
| Worry | 0.256 | 0.230 | 0.278 | 0.212 |
| Pain | 0.197 | 0.155 | 0.213 | 0.146 |
| Enjoyment | -0.162 | -0.090 | -0.153 | -0.178 |

Notes: All coefficients are Pearson’s correlation coefficients. All correlations are statistically significant at the 1% level. Data are from the Gallup World Poll (2023-2024), and sample weights are applied.

Table S4: Correlations of Loneliness with Well-Being and Emotional States by Region

| Correlation of lonely with… | Europe | Asia | Americas | MENA | SSA |
| --- | --- | --- | --- | --- | --- |
| SWB | -0.212 | -0.176 | -0.159 | -0.204 | -0.072 |
| Future SWB | -0.168 | -0.133 | -0.157 | -0.183 | -0.051 |
| Stress | 0.156 | 0.266 | 0.254 | 0.257 | 0.252 |
| Anger | 0.151 | 0.254 | 0.249 | 0.263 | 0.253 |
| Worry | 0.190 | 0.294 | 0.295 | 0.235 | 0.245 |
| Pain | 0.155 | 0.207 | 0.211 | 0.204 | 0.169 |
| Enjoyment | -0.177 | -0.167 | -0.195 | -0.149 | -0.096 |

Notes: All coefficients are Pearson’s correlation coefficients. All correlations are statistically significant at the 1% level. Data are from the Gallup World Poll (2023-2024), and sample weights are applied.

Table S5: Regional Differences in the Predictors of Loneliness

|  | Europe | Asia | Americas | MENA | SSA |
| --- | --- | --- | --- | --- | --- |
| Log(Income) | 0.017^***^ | -0.002 | 0.014^***^ | 0.008 | -0.006 |
|  | [0.009,0.026] | [-0.009,0.005] | [0.007,0.021] | [-0.014,0.030] | [-0.014,0.003] |
| Log(Income) Squared | -0.002^***^ | -0.001^**^ | -0.002^***^ | -0.002^*^ | -0.000 |
|  | [-0.003,-0.001] | [-0.001,-0.000] | [-0.003,-0.002] | [-0.004,0.000] | [-0.001,0.001] |
| Age | 0.001 | 0.004^***^ | 0.006^***^ | 0.005^***^ | 0.002^**^ |
|  | [-0.001,0.002] | [0.003,0.005] | [0.005,0.008] | [0.001,0.009] | [0.000,0.005] |
| Age – Squared | -0.000 | -0.000^***^ | -0.000^***^ | -0.000^***^ | -0.000^**^ |
|  | [-0.000,0.000] | [-0.000,-0.000] | [-0.000,-0.000] | [-0.000,-0.000] | [-0.000,-0.000] |
| Male | 0.010^***^ | 0.011^*^ | 0.004 | -0.001 | 0.013^***^ |
|  | [0.003,0.017] | [-0.001,0.023] | [-0.008,0.016] | [-0.032,0.029] | [0.003,0.022] |
| Religion | 0.023^***^ | 0.018^***^ | 0.020^***^ | 0.003 | 0.017 |
|  | [0.015,0.030] | [0.006,0.029] | [0.010,0.031] | [-0.021,0.026] | [-0.004,0.038] |
| *Educational Status* |  |  |  |  |  |
| Secondary | -0.033^***^ | -0.037^***^ | -0.066^***^ | -0.044^**^ | 0.000 |
|  | [-0.047,-0.020] | [-0.055,-0.018] | [-0.081,-0.050] | [-0.079,-0.010] | [-0.015,0.015] |
| Post-Secondary | -0.045^***^ | -0.050^***^ | -0.088^***^ | -0.086^***^ | -0.014 |
|  | [-0.057,-0.033] | [-0.071,-0.030] | [-0.108,-0.068] | [-0.133,-0.039] | [-0.040,0.013] |
| *Marital Status* |  |  |  |  |  |
| Married/Partner | -0.105^***^ | -0.089^***^ | -0.100^***^ | -0.078^***^ | -0.046^***^ |
|  | [-0.124,-0.087] | [-0.115,-0.064] | [-0.112,-0.088] | [-0.129,-0.027] | [-0.063,-0.029] |
| Separated/Divorced/Widowed | 0.077^***^ | 0.098^***^ | 0.060^***^ | 0.158^***^ | 0.081^***^ |
|  | [0.053,0.101] | [0.074,0.122] | [0.041,0.079] | [0.054,0.262] | [0.061,0.101] |
| Household Size | -0.027^***^ | -0.018^***^ | -0.021^***^ | -0.010^***^ | -0.004^***^ |
|  | [-0.034,-0.021] | [-0.022,-0.013] | [-0.027,-0.015] | [-0.013,-0.006] | [-0.005,-0.002] |
| Children | 0.016^**^ | 0.003 | -0.006 | -0.013 | -0.025^***^ |
|  | [0.003,0.029] | [-0.005,0.011] | [-0.019,0.006] | [-0.045,0.019] | [-0.040,-0.010] |
| Health Problem | 0.083^***^ | 0.103^***^ | 0.119^***^ | 0.122^***^ | 0.100^***^ |
|  | [0.072,0.094] | [0.093,0.113] | [0.106,0.132] | [0.080,0.165] | [0.085,0.115] |
| Immigrant | 0.020^***^ | 0.025^*^ | 0.050^***^ | 0.054^***^ | 0.019^**^ |
|  | [0.009,0.031] | [-0.002,0.051] | [0.027,0.074] | [0.013,0.095] | [0.001,0.037] |
| Urban | -0.010^***^ | -0.009 | -0.012 | 0.000 | -0.004 |
|  | [-0.017,-0.003] | [-0.022,0.003] | [-0.027,0.004] | [-0.028,0.029] | [-0.016,0.008] |
| Social Capital | -0.097^***^ | -0.078^***^ | -0.115^***^ | -0.104^***^ | -0.056^***^ |
|  | [-0.114,-0.079] | [-0.105,-0.051] | [-0.132,-0.098] | [-0.146,-0.063] | [-0.068,-0.044] |
| Safe | -0.042^***^ | -0.038^***^ | -0.025^***^ | -0.046^***^ | -0.012 |
|  | [-0.051,-0.032] | [-0.046,-0.029] | [-0.032,-0.017] | [-0.078,-0.014] | [-0.026,0.003] |
| Confidence Government | -0.020^***^ | -0.030^***^ | -0.012^**^ | -0.013 | -0.007 |
|  | [-0.027,-0.013] | [-0.042,-0.018] | [-0.022,-0.003] | [-0.037,0.012] | [-0.020,0.007] |
| *Employment Status* |  |  |  |  |  |
| Out-of-Workforce | -0.025^***^ | -0.060^***^ | -0.077^***^ | -0.080^***^ | -0.081^***^ |
|  | [-0.040,-0.009] | [-0.082,-0.039] | [-0.101,-0.052] | [-0.108,-0.052] | [-0.099,-0.063] |
| Part-time employed | -0.033^***^ | -0.043^***^ | -0.056^***^ | -0.070^***^ | -0.031^***^ |
|  | [-0.050,-0.017] | [-0.069,-0.018] | [-0.084,-0.028] | [-0.108,-0.031] | [-0.054,-0.008] |
| Full-time employed | -0.045^***^ | -0.063^***^ | -0.080^***^ | -0.109^***^ | -0.061^***^ |
|  | [-0.060,-0.030] | [-0.084,-0.042] | [-0.107,-0.053] | [-0.155,-0.063] | [-0.081,-0.040] |
| Obs. | 62,213 | 55,960 | 36,178 | 9,844 | 53,853 |
| Pseudo R^2^ | 0.14 | 0.12 | 0.13 | 0.10 | 0.04 |

Notes: Table shows average marginal effects from logit regressions. All regressions include country and year fixed effects. Omitted category for education is elementary, marital status is single, and employment is unemployed. Standard errors clustered at the country level in parentheses. Significance levels: *: *p*<.1, **: *p*<.05, ***: *p*<.01.

Table S6: Control Variables for SWB Regressions

|  | Current SWB | Future SWB |
| --- | --- | --- |
| Log(Income) | -0.167^***^ | -0.025 |
|  | [-0.202,-0.132] | [-0.061,0.011] |
| Log(Income) – Squared | 0.028^***^ | 0.008^***^ |
|  | [0.025,0.032] | [0.004,0.011] |
| Age | -0.040^***^ | -0.004 |
|  | [-0.048,-0.033] | [-0.011,0.002] |
| Age – Squared | 0.000^***^ | -0.000^***^ |
|  | [0.000,0.000] | [-0.000,-0.000] |
| Male | -0.192^***^ | -0.112^***^ |
|  | [-0.229,-0.156] | [-0.139,-0.084] |
| Religion | 0.055^***^ | 0.105^***^ |
|  | [0.015,0.094] | [0.070,0.139] |
| *Educational Status* |  |  |
| Secondary | 0.298^***^ | 0.190^***^ |
|  | [0.240,0.356] | [0.137,0.243] |
| Post-Secondary | 0.572^***^ | 0.253^***^ |
|  | [0.503,0.641] | [0.190,0.317] |
| *Marital Status* |  |  |
| Married/Partner | 0.115^***^ | -0.049^***^ |
|  | [0.068,0.162] | [-0.083,-0.014] |
| Separated/Divorced/Widowed | -0.091^***^ | -0.035 |
|  | [-0.142,-0.039] | [-0.081,0.011] |
| Household Size | 0.037^***^ | 0.016^***^ |
|  | [0.026,0.049] | [0.007,0.025] |
| Children | 0.055^***^ | 0.103^***^ |
|  | [0.014,0.096] | [0.066,0.140] |
| Health Problem | -0.420^***^ | -0.239^***^ |
|  | [-0.473,-0.366] | [-0.270,-0.209] |
| Immigrant | -0.005 | 0.126^***^ |
|  | [-0.060,0.050] | [0.063,0.189] |
| Urban | 0.103^***^ | 0.055^***^ |
|  | [0.057,0.149] | [0.022,0.089] |
| Social Capital | 0.711^***^ | 0.259^***^ |
|  | [0.658,0.764] | [0.217,0.301] |
| Safe | 0.201^***^ | 0.120^***^ |
|  | [0.165,0.238] | [0.090,0.150] |
| Confidence Government | 0.391^***^ | 0.190^***^ |
|  | [0.349,0.432] | [0.150,0.230] |
| *Employment Status* |  |  |
| Out-of-Workforce | 0.395^***^ | -0.131^***^ |
|  | [0.332,0.458] | [-0.188,-0.075] |
| Part-time employed | 0.423^***^ | -0.009 |
|  | [0.348,0.498] | [-0.068,0.050] |
| Full-time employed | 0.408^***^ | -0.018 |
|  | [0.341,0.474] | [-0.084,0.048] |
| *Current SWB Levels* |  |  |
| 1 |  | -0.218^**^ |
|  |  | [-0.400,-0.036] |
| 2 |  | 0.092 |
|  |  | [-0.104,0.289] |
| 3 |  | 0.630^***^ |
|  |  | [0.392,0.867] |
| 4 |  | 1.225^***^ |
|  |  | [0.966,1.484] |
| 5 |  | 1.925^***^ |
|  |  | [1.643,2.207] |
| 6 |  | 2.494^***^ |
|  |  | [2.201,2.787] |
| 7 |  | 3.161^***^ |
|  |  | [2.851,3.471] |
| 8 |  | 3.755^***^ |
|  |  | [3.432,4.079] |
| 9 |  | 4.199^***^ |
|  |  | [3.844,4.555] |
| 10 |  | 4.057^***^ |
|  |  | [3.671,4.444] |
| Obs. | 216,591 | 203,739 |
| Adj. *R*^2^ | 0.25 | 0.38 |

Notes: All regressions include country and year fixed effects. Standard errors clustered at the country level and 95% Confidence intervals in parentheses. Significance levels: *: *p*<.1, **: *p*<.05, ***: *p*<.01.

Table S7: Control Variables for SWB Regression by World Bank Development Group

|  | **Current SWB** | | | **Future SWB** | | | |  |
| --- | --- | --- | --- | --- | --- | --- | --- | --- |
|  | Low | Middle | High | | Low | Middle | High | |
| Log(Income) | -0.077^*^ | -0.230^***^ | -0.264^***^ | | 0.034 | -0.063^*^ | -0.051^***^ | |
|  | [-0.163,0.009] | [-0.277,-0.184] | [-0.312,-0.216] | | [-0.096,0.163] | [-0.125,0.000] | [-0.088,-0.015] | |
| Log(Income) Squared | 0.024^***^ | 0.037^***^ | 0.031^***^ | | 0.006 | 0.013^***^ | 0.005^***^ | |
|  | [0.009,0.039] | [0.031,0.043] | [0.026,0.036] | | [-0.013,0.024] | [0.006,0.019] | [0.003,0.008] | |
| Age | -0.024^**^ | -0.039^***^ | -0.041^***^ | | -0.006 | -0.009^*^ | 0.001 | |
|  | [-0.045,-0.003] | [-0.051,-0.028] | [-0.050,-0.032] | | [-0.021,0.010] | [-0.018,0.000] | [-0.008,0.010] | |
| Age – Squared | 0.000^**^ | 0.000^***^ | 0.000^***^ | | -0.000 | -0.000^***^ | -0.000^***^ | |
|  | [0.000,0.001] | [0.000,0.000] | [0.000,0.001] | | [-0.000,0.000] | [-0.000,-0.000] | [-0.000,-0.000] | |
| Male | -0.147^***^ | -0.247^***^ | -0.151^***^ | | -0.028 | -0.169^***^ | -0.060^***^ | |
|  | [-0.225,-0.069] | [-0.307,-0.187] | [-0.190,-0.112] | | [-0.114,0.058] | [-0.214,-0.124] | [-0.083,-0.037] | |
| Religion | -0.191^***^ | 0.087^***^ | 0.051^*^ | | 0.238^*^ | 0.140^***^ | 0.053^**^ | |
|  | [-0.309,-0.072] | [0.032,0.142] | [-0.004,0.107] | | [-0.011,0.486] | [0.085,0.195] | [0.013,0.092] | |
| *Educational Status* |  |  |  | |  |  |  | |
| Secondary | 0.446^***^ | 0.224^***^ | 0.253^***^ | | 0.360^***^ | 0.156^***^ | 0.101^*^ | |
|  | [0.276,0.615] | [0.150,0.298] | [0.158,0.348] | | [0.283,0.438] | [0.091,0.221] | [-0.007,0.209] | |
| Post-Secondary | 1.131^***^ | 0.500^***^ | 0.488^***^ | | 0.652^***^ | 0.198^***^ | 0.140^**^ | |
|  | [0.794,1.468] | [0.407,0.594] | [0.379,0.596] | | [0.419,0.886] | [0.111,0.285] | [0.028,0.253] | |
| *Marital Status* |  |  |  | |  |  |  | |
| Married/Partner | -0.100 | 0.034 | 0.255^***^ | | 0.041 | -0.049^*^ | -0.089^***^ | |
|  | [-0.252,0.053] | [-0.030,0.099] | [0.211,0.300] | | [-0.067,0.150] | [-0.101,0.004] | [-0.135,-0.043] | |
| Separated/Divorced/Widowed | -0.433^***^ | -0.093^**^ | 0.015 | | -0.190^**^ | 0.005 | -0.053^*^ | |
|  | [-0.579,-0.287] | [-0.173,-0.014] | [-0.032,0.061] | | [-0.341,-0.039] | [-0.067,0.078] | [-0.116,0.010] | |
| Household Size | 0.016 | 0.049^***^ | 0.061^***^ | | 0.016 | 0.025^***^ | -0.008 | |
|  | [-0.010,0.043] | [0.036,0.062] | [0.040,0.083] | | [-0.010,0.042] | [0.013,0.038] | [-0.020,0.005] | |
| Children | 0.063 | 0.031 | 0.077^***^ | | 0.007 | 0.107^***^ | 0.114^***^ | |
|  | [-0.126,0.252] | [-0.024,0.085] | [0.040,0.115] | | [-0.166,0.179] | [0.054,0.160] | [0.068,0.160] | |
| Health Problem | -0.149 | -0.362^***^ | -0.557^***^ | | -0.168^**^ | -0.240^***^ | -0.172^***^ | |
|  | [-0.333,0.035] | [-0.431,-0.292] | [-0.613,-0.501] | | [-0.294,-0.041] | [-0.283,-0.196] | [-0.222,-0.122] | |
| Immigrant | 0.108 | 0.084 | -0.045 | | 0.181^**^ | 0.070 | 0.134^***^ | |
|  | [-0.055,0.270] | [-0.018,0.187] | [-0.112,0.022] | | [0.018,0.344] | [-0.040,0.180] | [0.065,0.203] | |
| Urban | 0.060 | 0.156^***^ | 0.009 | | 0.090 | 0.065^**^ | 0.028^*^ | |
|  | [-0.053,0.174] | [0.083,0.229] | [-0.028,0.047] | | [-0.111,0.291] | [0.014,0.117] | [-0.002,0.058] | |
| Social Capital | 0.648^***^ | 0.706^***^ | 0.695^***^ | | 0.159^**^ | 0.255^***^ | 0.271^***^ | |
|  | [0.528,0.768] | [0.633,0.780] | [0.614,0.776] | | [0.045,0.273] | [0.198,0.312] | [0.213,0.329] | |
| Safe | 0.090^*^ | 0.152^***^ | 0.349^***^ | | 0.058 | 0.091^***^ | 0.157^***^ | |
|  | [-0.020,0.200] | [0.107,0.197] | [0.295,0.402] | | [-0.045,0.162] | [0.051,0.132] | [0.128,0.186] | |
| Confidence Government | 0.291^***^ | 0.388^***^ | 0.424^***^ | | 0.223^***^ | 0.193^***^ | 0.136^***^ | |
|  | [0.149,0.433] | [0.328,0.447] | [0.362,0.487] | | [0.081,0.366] | [0.133,0.253] | [0.086,0.186] | |
| *Employment Status* |  |  |  | |  |  |  | |
| Out-of-Workforce | 0.219^**^ | 0.464^***^ | 0.364^***^ | | -0.086 | -0.096^**^ | -0.265^***^ | |
|  | [0.047,0.390] | [0.382,0.546] | [0.262,0.466] | | [-0.278,0.105] | [-0.168,-0.024] | [-0.362,-0.167] | |
| Part-time employed | 0.119^**^ | 0.466^***^ | 0.504^***^ | | 0.037 | 0.006 | -0.128^***^ | |
|  | [0.003,0.234] | [0.358,0.574] | [0.401,0.608] | | [-0.153,0.227] | [-0.079,0.090] | [-0.208,-0.048] | |
| Full-time employed | 0.221^***^ | 0.452^***^ | 0.410^***^ | | 0.108 | 0.005 | -0.157^***^ | |
|  | [0.089,0.354] | [0.354,0.551] | [0.318,0.502] | | [-0.058,0.275] | [-0.095,0.105] | [-0.254,-0.061] | |
| *Current SWB Levels* |  |  |  | |  |  |  | |
| 1 |  |  |  | | -0.247 | -0.232^*^ | -0.124 | |
|  |  |  |  | | [-0.593,0.098] | [-0.471,0.006] | [-0.593,0.345] | |
| 2 |  |  |  | | -0.005 | 0.118 | 0.470^***^ | |
|  |  |  |  | | [-0.402,0.392] | [-0.129,0.365] | [0.124,0.816] | |
| 3 |  |  |  | | 0.628^**^ | 0.684^***^ | 0.939^***^ | |
|  |  |  |  | | [0.172,1.084] | [0.377,0.991] | [0.537,1.340] | |
| 4 |  |  |  | | 1.091^***^ | 1.315^***^ | 1.726^***^ | |
|  |  |  |  | | [0.604,1.578] | [0.982,1.648] | [1.345,2.107] | |
| 5 |  |  |  | | 1.689^***^ | 2.018^***^ | 2.492^***^ | |
|  |  |  |  | | [1.237,2.142] | [1.651,2.384] | [2.088,2.896] | |
| 6 |  |  |  | | 2.131^***^ | 2.522^***^ | 3.322^***^ | |
|  |  |  |  | | [1.684,2.577] | [2.143,2.900] | [2.892,3.753] | |
| 7 |  |  |  | | 2.378^***^ | 3.172^***^ | 4.067^***^ | |
|  |  |  |  | | [1.846,2.910] | [2.769,3.576] | [3.619,4.514] | |
| 8 |  |  |  | | 2.528^***^ | 3.678^***^ | 4.764^***^ | |
|  |  |  |  | | [2.012,3.044] | [3.249,4.107] | [4.298,5.229] | |
| 9 |  |  |  | | 2.364^***^ | 3.843^***^ | 5.425^***^ | |
|  |  |  |  | | [1.698,3.030] | [3.355,4.331] | [4.941,5.909] | |
| 10 |  |  |  | | 2.680^***^ | 3.860^***^ | 5.835^***^ | |
|  |  |  |  | | [2.116,3.243] | [3.384,4.336] | [5.228,6.443] | |
| Obs. | 21,534 | 115,844 | 77,817 | | 20,569 | 106,675 | 75,225 | |
| Adj. *R*^2^ | 0.09 | 0.18 | 0.21 | | 0.22 | 0.36 | 0.52 | |

Notes: All regressions include country and year fixed effects. Standard errors clustered at the country level and 95% Confidence intervals in parentheses. Significance levels: *: *p*<.1, **: *p*<.05, ***: *p*<.01.

Table S8: Predictors of Current SWB by Region

|  | Europe | Asia | Americas | MENA | SSA |
| --- | --- | --- | --- | --- | --- |
| Lonely | -0.635^***^ | -0.515^***^ | -0.561^***^ | -0.684^***^ | -0.229^***^ |
|  | [-0.722,-0.547] | [-0.629,-0.401] | [-0.671,-0.451] | [-1.111,-0.257] | [-0.326,-0.132] |
| Log(Income) | -0.299^***^ | -0.220^***^ | -0.213^***^ | -0.255^*^ | -0.118^***^ |
|  | [-0.371,-0.228] | [-0.282,-0.157] | [-0.290,-0.136] | [-0.539,0.029] | [-0.164,-0.072] |
| Log(Income) Squared | 0.035^***^ | 0.034^***^ | 0.030^***^ | 0.043^**^ | 0.027^***^ |
|  | [0.028,0.043] | [0.028,0.040] | [0.020,0.039] | [0.012,0.075] | [0.020,0.033] |
| Age | -0.038^***^ | -0.037^***^ | -0.060^***^ | -0.059^**^ | -0.040^***^ |
|  | [-0.045,-0.031] | [-0.053,-0.020] | [-0.076,-0.044] | [-0.101,-0.017] | [-0.056,-0.024] |
| Age – Squared | 0.000^***^ | 0.000^***^ | 0.001^***^ | 0.001^**^ | 0.000^***^ |
|  | [0.000,0.000] | [0.000,0.001] | [0.000,0.001] | [0.000,0.001] | [0.000,0.001] |
| Male | -0.135^***^ | -0.280^***^ | -0.288^***^ | -0.342^*^ | -0.126^***^ |
|  | [-0.183,-0.086] | [-0.358,-0.202] | [-0.395,-0.182] | [-0.766,0.081] | [-0.173,-0.080] |
| Religion | 0.031 | 0.085^**^ | 0.109^***^ | 0.446^*^ | -0.097^*^ |
|  | [-0.018,0.081] | [0.011,0.159] | [0.033,0.186] | [-0.024,0.916] | [-0.197,0.002] |
| *Educational Status* |  |  |  |  |  |
| Secondary | 0.261^***^ | 0.161^*^ | 0.229^***^ | 0.346^***^ | 0.383^***^ |
|  | [0.161,0.362] | [-0.012,0.335] | [0.090,0.368] | [0.163,0.528] | [0.289,0.476] |
| Post-Secondary | 0.481^***^ | 0.438^***^ | 0.484^***^ | 0.658^**^ | 0.789^***^ |
|  | [0.356,0.607] | [0.272,0.605] | [0.315,0.654] | [0.214,1.102] | [0.611,0.966] |
| *Marital Status* |  |  |  |  |  |
| Married/Partner | 0.182^***^ | 0.108^*^ | 0.089^*^ | 0.091 | 0.019 |
|  | [0.132,0.231] | [-0.012,0.227] | [-0.010,0.189] | [-0.315,0.498] | [-0.081,0.120] |
| Separated/Divorced/Widowed | -0.022 | -0.084^*^ | 0.031 | -0.093 | -0.217^***^ |
|  | [-0.070,0.027] | [-0.182,0.013] | [-0.072,0.134] | [-0.610,0.424] | [-0.362,-0.071] |
| Household Size | 0.082^***^ | 0.052^***^ | 0.055^***^ | 0.091^***^ | 0.024^***^ |
|  | [0.056,0.108] | [0.029,0.075] | [0.037,0.073] | [0.056,0.126] | [0.009,0.040] |
| Children | 0.067^***^ | 0.023 | -0.019 | 0.048 | 0.012 |
|  | [0.018,0.116] | [-0.050,0.096] | [-0.079,0.041] | [-0.036,0.133] | [-0.095,0.120] |
| Health Problem | -0.550^***^ | -0.460^***^ | -0.525^***^ | -0.417^***^ | -0.151^***^ |
|  | [-0.610,-0.490] | [-0.552,-0.368] | [-0.630,-0.420] | [-0.636,-0.198] | [-0.263,-0.040] |
| Immigrant | -0.042 | -0.006 | -0.134 | -0.057 | 0.210^***^ |
|  | [-0.104,0.020] | [-0.121,0.110] | [-0.325,0.058] | [-0.203,0.089] | [0.083,0.337] |
| Urban | 0.050^**^ | 0.100 | 0.133^***^ | 0.118 | 0.142^**^ |
|  | [0.006,0.094] | [-0.029,0.229] | [0.036,0.229] | [-0.222,0.458] | [0.030,0.254] |
| Social Capital | 0.734^***^ | 0.761^***^ | 0.716^***^ | 0.630^***^ | 0.640^***^ |
|  | [0.624,0.845] | [0.654,0.869] | [0.610,0.822] | [0.454,0.807] | [0.544,0.736] |
| Safe | 0.314^***^ | 0.176^***^ | 0.243^***^ | 0.194 | 0.091^**^ |
|  | [0.252,0.377] | [0.102,0.250] | [0.185,0.300] | [-0.091,0.480] | [0.022,0.160] |
| Confidence Government | 0.373^***^ | 0.444^***^ | 0.401^***^ | 0.410^***^ | 0.345^***^ |
|  | [0.301,0.444] | [0.352,0.535] | [0.313,0.488] | [0.256,0.565] | [0.251,0.439] |
| *Employment Status* |  |  |  |  |  |
| Out-of-Workforce | 0.380^***^ | 0.446^***^ | 0.560^***^ | 0.667^***^ | 0.266^***^ |
|  | [0.268,0.492] | [0.343,0.548] | [0.431,0.689] | [0.341,0.992] | [0.162,0.370] |
| Part-time employed | 0.510^***^ | 0.409^***^ | 0.607^***^ | 0.567^***^ | 0.261^***^ |
|  | [0.393,0.628] | [0.209,0.608] | [0.454,0.760] | [0.321,0.813] | [0.153,0.370] |
| Full-time employed | 0.415^***^ | 0.364^***^ | 0.589^***^ | 0.655^***^ | 0.291^***^ |
|  | [0.309,0.521] | [0.217,0.511] | [0.429,0.749] | [0.504,0.806] | [0.182,0.401] |
| Obs. | 62,052 | 55,486 | 35,851 | 9,826 | 53,376 |
| Adj. *R*^2^ | 0.24 | 0.22 | 0.12 | 0.25 | 0.09 |

Notes: All regressions include country and year fixed effects. Standard errors clustered at the country level and 95% Confidence intervals in parentheses. Significance levels: *: *p*<.1, **: *p*<.05, ***: *p*<.01.

Table S9: Predictors of Future SWB by Region

|  | Europe | Asia | Americas | MENA | SSA |
| --- | --- | --- | --- | --- | --- |
| Lonely | -0.050^**^ | -0.057^*^ | -0.104^**^ | -0.137 | -0.044^*^ |
|  | [-0.096,-0.003] | [-0.115,0.001] | [-0.181,-0.027] | [-0.359,0.085] | [-0.093,0.004] |
| *Current SWB Levels* |  |  |  |  |  |
| 1 | 0.057 | 0.255 | -0.664^***^ | 0.065 | -0.298^**^ |
|  | [-0.353,0.466] | [-0.134,0.644] | [-0.988,-0.340] | [-0.770,0.900] | [-0.530,-0.066] |
| 2 | 0.700^***^ | 0.519^***^ | -0.271^*^ | 0.477 | 0.030 |
|  | [0.382,1.018] | [0.187,0.851] | [-0.557,0.016] | [-0.371,1.326] | [-0.259,0.319] |
| 3 | 1.309^***^ | 1.340^***^ | 0.043 | 1.324^***^ | 0.555^***^ |
|  | [0.933,1.685] | [0.939,1.742] | [-0.271,0.357] | [0.604,2.045] | [0.231,0.879] |
| 4 | 2.201^***^ | 1.991^***^ | 0.726^***^ | 1.869^***^ | 1.067^***^ |
|  | [1.834,2.567] | [1.558,2.423] | [0.398,1.053] | [0.945,2.793] | [0.708,1.426] |
| 5 | 2.955^***^ | 2.846^***^ | 1.505^***^ | 2.688^***^ | 1.630^***^ |
|  | [2.580,3.330] | [2.396,3.297] | [1.207,1.802] | [1.897,3.479] | [1.257,2.004] |
| 6 | 3.799^***^ | 3.456^***^ | 2.109^***^ | 3.222^***^ | 1.991^***^ |
|  | [3.411,4.188] | [2.994,3.917] | [1.802,2.416] | [2.383,4.062] | [1.621,2.360] |
| 7 | 4.592^***^ | 4.125^***^ | 2.686^***^ | 3.944^***^ | 2.402^***^ |
|  | [4.201,4.984] | [3.615,4.636] | [2.366,3.005] | [3.048,4.840] | [1.999,2.805] |
| 8 | 5.301^***^ | 4.788^***^ | 3.168^***^ | 4.522^***^ | 2.610^***^ |
|  | [4.901,5.700] | [4.272,5.305] | [2.840,3.496] | [3.683,5.362] | [2.150,3.070] |
| 9 | 5.929^***^ | 5.285^***^ | 3.521^***^ | 4.975^***^ | 2.519^***^ |
|  | [5.524,6.334] | [4.745,5.824] | [3.094,3.949] | [4.030,5.921] | [1.958,3.080] |
| 10 | 6.434^***^ | 5.273^***^ | 3.463^***^ | 5.024^***^ | 2.785^***^ |
|  | [6.003,6.866] | [4.659,5.888] | [3.043,3.883] | [4.101,5.948] | [2.371,3.199] |
| Log(Income) | -0.080^***^ | -0.009 | 0.001 | -0.155^*^ | -0.037 |
|  | [-0.121,-0.039] | [-0.061,0.043] | [-0.049,0.050] | [-0.318,0.009] | [-0.138,0.064] |
| Log(Income) Squared | 0.008^***^ | 0.007^***^ | 0.003 | 0.019^**^ | 0.011^**^ |
|  | [0.005,0.011] | [0.002,0.013] | [-0.002,0.008] | [0.006,0.033] | [0.001,0.022] |
| Age | -0.002 | -0.009 | -0.017^**^ | -0.029 | -0.007 |
|  | [-0.015,0.011] | [-0.021,0.003] | [-0.030,-0.004] | [-0.070,0.012] | [-0.017,0.004] |
| Age – Squared | -0.000^***^ | -0.000^***^ | -0.000^**^ | 0.000 | -0.000^*^ |
|  | [-0.000,-0.000] | [-0.000,-0.000] | [-0.000,-0.000] | [-0.000,0.000] | [-0.000,0.000] |
| Male | -0.049^***^ | -0.148^***^ | -0.216^***^ | -0.146^*^ | -0.059^*^ |
|  | [-0.078,-0.020] | [-0.216,-0.080] | [-0.264,-0.168] | [-0.318,0.027] | [-0.123,0.004] |
| Religion | 0.060^**^ | 0.144^***^ | 0.094^**^ | -0.049 | 0.231^***^ |
|  | [0.013,0.107] | [0.073,0.215] | [0.011,0.177] | [-0.266,0.169] | [0.118,0.343] |
| *Educational Status* |  |  |  |  |  |
| Secondary | 0.101^**^ | 0.021 | 0.277^***^ | 0.113 | 0.282^***^ |
|  | [0.020,0.182] | [-0.090,0.131] | [0.159,0.395] | [-0.119,0.346] | [0.208,0.357] |
| Post-Secondary | 0.143^***^ | 0.043 | 0.343^***^ | 0.137 | 0.464^***^ |
|  | [0.056,0.231] | [-0.081,0.168] | [0.203,0.482] | [-0.141,0.414] | [0.284,0.644] |
| *Marital Status* |  |  |  |  |  |
| Married/Partner | -0.150^***^ | -0.005 | -0.084^**^ | -0.141^*^ | 0.047 |
|  | [-0.202,-0.098] | [-0.068,0.057] | [-0.159,-0.009] | [-0.301,0.019] | [-0.031,0.124] |
| Separated/Divorced/Widowed | -0.078^**^ | 0.020 | 0.004 | -0.046 | -0.054 |
|  | [-0.148,-0.009] | [-0.082,0.122] | [-0.086,0.095] | [-0.328,0.237] | [-0.188,0.079] |
| Household Size | 0.002 | 0.022^**^ | 0.017^*^ | 0.034^***^ | 0.018^**^ |
|  | [-0.016,0.019] | [0.004,0.040] | [-0.003,0.036] | [0.018,0.050] | [0.003,0.033] |
| Children | 0.083^***^ | 0.150^***^ | 0.092^**^ | -0.119 | 0.012 |
|  | [0.036,0.130] | [0.089,0.210] | [0.022,0.163] | [-0.436,0.199] | [-0.068,0.091] |
| Health Problem | -0.149^***^ | -0.204^***^ | -0.307^***^ | -0.342^***^ | -0.179^***^ |
|  | [-0.201,-0.098] | [-0.267,-0.140] | [-0.372,-0.241] | [-0.517,-0.167] | [-0.251,-0.107] |
| Immigrant | 0.161^***^ | 0.091^**^ | 0.237^***^ | -0.023 | 0.019 |
|  | [0.051,0.272] | [0.005,0.176] | [0.070,0.403] | [-0.172,0.127] | [-0.128,0.167] |
| Urban | 0.028^*^ | 0.020 | 0.068^*^ | 0.069 | 0.115^**^ |
|  | [-0.005,0.061] | [-0.038,0.079] | [-0.010,0.145] | [-0.217,0.354] | [0.017,0.214] |
| Social Capital | 0.266^***^ | 0.294^***^ | 0.188^***^ | 0.387^***^ | 0.201^***^ |
|  | [0.197,0.335] | [0.232,0.356] | [0.118,0.258] | [0.198,0.575] | [0.113,0.289] |
| Safe | 0.155^***^ | 0.083^**^ | 0.122^***^ | 0.105^**^ | 0.072^**^ |
|  | [0.108,0.203] | [0.012,0.153] | [0.065,0.178] | [0.015,0.194] | [0.006,0.138] |
| Confidence Government | 0.126^***^ | 0.181^***^ | 0.233^***^ | 0.336^***^ | 0.174^***^ |
|  | [0.066,0.186] | [0.101,0.262] | [0.146,0.319] | [0.224,0.447] | [0.083,0.265] |
| *Employment Status* |  |  |  |  |  |
| Out-of-Workforce | -0.235^***^ | -0.159^***^ | -0.204^**^ | 0.186^*^ | -0.098^**^ |
|  | [-0.330,-0.141] | [-0.270,-0.047] | [-0.355,-0.052] | [-0.030,0.402] | [-0.190,-0.006] |
| Part-time employed | -0.143^***^ | -0.047 | -0.005 | 0.153 | 0.009 |
|  | [-0.234,-0.051] | [-0.172,0.077] | [-0.135,0.125] | [-0.189,0.495] | [-0.101,0.119] |
| Full-time employed | -0.147^***^ | -0.116 | 0.060 | 0.269 | 0.032 |
|  | [-0.250,-0.045] | [-0.263,0.031] | [-0.098,0.219] | [-0.093,0.631] | [-0.065,0.129] |
| Obs. | 59,505 | 51,062 | 32,930 | 9,048 | 51,194 |
| Adj. *R*^2^ | 0.55 | 0.46 | 0.36 | 0.43 | 0.22 |

Notes: All regressions include country and year fixed effects. Standard errors clustered at the country level and 95% Confidence intervals in parentheses. Significance levels: *: *p*<.1, **: *p*<.05, ***: *p*<.01.

Table S10: Controls for Emotion Regressions

|  | Stress | Anger | Worry | Pain | Enjoyment | Migration Intention |
| --- | --- | --- | --- | --- | --- | --- |
| Log(Income) | 0.001 | 0.002 | 0.010^***^ | 0.013^***^ | -0.010^***^ | 0.000 |
|  | [-0.004,0.005] | [-0.002,0.005] | [0.005,0.015] | [0.008,0.017] | [-0.014,-0.005] | [-0.004,0.005] |
| Log(Income) Squared | -0.001^***^ | -0.001^***^ | -0.002^***^ | -0.002^***^ | 0.002^***^ | -0.000 |
|  | [-0.001,-0.000] | [-0.001,-0.000] | [-0.002,-0.001] | [-0.002,-0.002] | [0.001,0.002] | [-0.000,0.000] |
| Age | 0.007^***^ | 0.001^***^ | 0.008^***^ | 0.003^***^ | -0.004^***^ | -0.002^***^ |
|  | [0.006,0.008] | [0.001,0.002] | [0.006,0.009] | [0.003,0.004] | [-0.006,-0.003] | [-0.003,-0.001] |
| Age – Squared | -0.000^***^ | -0.000^***^ | -0.000^***^ | -0.000^***^ | 0.000^***^ | -0.000^***^ |
|  | [-0.000,-0.000] | [-0.000,-0.000] | [-0.000,-0.000] | [-0.000,-0.000] | [0.000,0.000] | [-0.000,-0.000] |
| Male | -0.034^***^ | -0.015^***^ | -0.032^***^ | -0.035^***^ | -0.010^***^ | 0.020^***^ |
|  | [-0.043,-0.026] | [-0.020,-0.009] | [-0.039,-0.025] | [-0.043,-0.028] | [-0.016,-0.004] | [0.013,0.026] |
| Religion | -0.005 | -0.002 | 0.010^**^ | 0.019^***^ | 0.043^***^ | -0.021^***^ |
|  | [-0.014,0.004] | [-0.008,0.004] | [0.001,0.018] | [0.013,0.025] | [0.036,0.049] | [-0.030,-0.012] |
| *Educational Status* |  |  |  |  |  |  |
| Secondary | -0.023^***^ | -0.016^***^ | -0.011^**^ | -0.026^***^ | 0.017^***^ | 0.051^***^ |
|  | [-0.032,-0.014] | [-0.022,-0.009] | [-0.023,-0.000] | [-0.033,-0.018] | [0.006,0.027] | [0.042,0.060] |
| Post-Secondary | -0.002 | -0.025^***^ | -0.000 | -0.060^***^ | 0.030^***^ | 0.055^***^ |
|  | [-0.013,0.009] | [-0.033,-0.017] | [-0.014,0.013] | [-0.070,-0.050] | [0.018,0.042] | [0.043,0.067] |
| *Marital Status* |  |  |  |  |  |  |
| Married/Partner | 0.014^***^ | 0.016^***^ | 0.023^***^ | 0.023^***^ | 0.013^***^ | -0.039^***^ |
|  | [0.006,0.021] | [0.010,0.021] | [0.016,0.031] | [0.017,0.029] | [0.006,0.020] | [-0.046,-0.032] |
| Separated/Divorced/Widowed | -0.002 | -0.008^**^ | 0.009^**^ | 0.014^***^ | 0.002 | -0.025^***^ |
|  | [-0.010,0.007] | [-0.015,-0.001] | [0.000,0.018] | [0.007,0.021] | [-0.006,0.010] | [-0.034,-0.017] |
| Household Size | 0.002^**^ | 0.002^***^ | 0.001 | 0.000 | 0.001 | -0.002^**^ |
|  | [0.000,0.003] | [0.001,0.003] | [-0.001,0.002] | [-0.001,0.001] | [-0.000,0.003] | [-0.003,-0.000] |
| Children | 0.011^***^ | 0.014^***^ | 0.002 | -0.000 | 0.004 | 0.007^**^ |
|  | [0.004,0.018] | [0.009,0.020] | [-0.005,0.008] | [-0.007,0.006] | [-0.004,0.011] | [0.001,0.013] |
| Health Problem | 0.111^***^ | 0.053^***^ | 0.147^***^ | 0.261^***^ | -0.052^***^ | 0.004 |
|  | [0.102,0.121] | [0.047,0.059] | [0.138,0.157] | [0.246,0.276] | [-0.060,-0.044] | [-0.002,0.011] |
| Immigrant | -0.001 | -0.002 | 0.001 | 0.001 | -0.011^*^ | 0.044^***^ |
|  | [-0.012,0.010] | [-0.011,0.008] | [-0.014,0.015] | [-0.008,0.011] | [-0.024,0.001] | [0.028,0.060] |
| Urban | 0.012^***^ | 0.008^***^ | 0.003 | -0.008^***^ | -0.002 | 0.030^***^ |
|  | [0.007,0.018] | [0.004,0.012] | [-0.004,0.010] | [-0.013,-0.003] | [-0.008,0.005] | [0.023,0.036] |
| Social Capital | -0.062^***^ | -0.041^***^ | -0.077^***^ | -0.045^***^ | 0.108^***^ | -0.024^***^ |
|  | [-0.070,-0.053] | [-0.048,-0.034] | [-0.086,-0.068] | [-0.050,-0.039] | [0.099,0.117] | [-0.031,-0.016] |
| Safe | -0.047^***^ | -0.029^***^ | -0.051^***^ | -0.025^***^ | 0.051^***^ | -0.046^***^ |
|  | [-0.054,-0.040] | [-0.034,-0.023] | [-0.060,-0.043] | [-0.030,-0.019] | [0.045,0.057] | [-0.052,-0.039] |
| Confidence Government | -0.055^***^ | -0.041^***^ | -0.057^***^ | -0.031^***^ | 0.065^***^ | -0.121^***^ |
|  | [-0.062,-0.048] | [-0.047,-0.035] | [-0.064,-0.051] | [-0.037,-0.025] | [0.058,0.072] | [-0.132,-0.110] |
| *Employment Status* |  |  |  |  |  |  |
| Out-of-Workforce | -0.075^***^ | -0.034^***^ | -0.092^***^ | 0.002 | 0.016^***^ | -0.066^***^ |
|  | [-0.087,-0.064] | [-0.043,-0.025] | [-0.102,-0.081] | [-0.007,0.011] | [0.005,0.027] | [-0.077,-0.056] |
| Part-time employed | -0.031^***^ | -0.010^**^ | -0.062^***^ | 0.017^***^ | 0.061^***^ | -0.034^***^ |
|  | [-0.043,-0.019] | [-0.019,-0.000] | [-0.073,-0.051] | [0.006,0.028] | [0.050,0.071] | [-0.045,-0.023] |
| Full-time employed | -0.013^**^ | -0.016^***^ | -0.063^***^ | 0.019^***^ | 0.041^***^ | -0.050^***^ |
|  | [-0.026,-0.001] | [-0.024,-0.009] | [-0.073,-0.054] | [0.008,0.029] | [0.029,0.053] | [-0.061,-0.040] |
| Obs. | 217,445 | 217,580 | 217,609 | 217,790 | 216,928 | 215,508 |
| Pseudo R^2^ | 0.11 | 0.10 | 0.11 | 0.14 | 0.10 | 0.18 |

Notes: Table shows average marginal effects from logit regressions. All regressions include country and year fixed effects. Standard errors clustered at the country level and 95% Confidence intervals in parentheses. Significance levels: *: *p*<.1, **: *p*<.05, ***: *p*<.01.

Figure S1: Change in Country-Level Prevalence of Loneliness

Notes: The figure displays the change in the prevalence of loneliness from 2023 to 2024 by country and World Bank development group. Bars represent percentage point changes. Data are based on Gallup World Poll. Analysis includes only countries surveyed in both years (*N*=113). Sample weights applied.

Figure S2: Differences in the Association Between Loneliness and Emotional/Behavioral Outcomes by Development Group

Notes: Table shows average marginal effects from logit regressions. All regressions include all controls, country and year fixed effects. Standard errors clustered at the country level. 95% Confidence intervals shown.

Figure S3: Regional Differences in the Association Between Loneliness and Emotional/Behavioral Outcomes

Notes: Table shows average marginal effects from logit regressions. All regressions include all controls, country and year fixed effects. Standard errors clustered at the country level. 95% Confidence intervals shown.
